# SFXN2 contributes mitochondrial dysfunction-induced apoptosis as a substrate of Parkin

**DOI:** 10.3389/fncel.2025.1623747

**Published:** 2025-08-14

**Authors:** Shishi Luo, Yechuan He, Yaohui He, Danling Wang

**Affiliations:** ^1^Hengyang Medical School, University of South China, Hengyang, China; ^2^Institute for Future Sciences, University of South China, Changsha, China; ^3^MOE Key Lab of Rare Pediatric Diseases, School of Life Sciences, University of South China, Changsha, China; ^4^Department of Pathology, The Sixth Affiliated Hospital, Sun Yat-sen University, Guangzhou, China; ^5^Biomedical Innovation Center, The Sixth Affiliated Hospital, Sun Yat-sen University, Guangzhou, China

**Keywords:** SFXN2, Parkin, ubiquitination, apoptosis, mitochondrial dysfunction

## Abstract

**Introduction:**

Mitochondria, situated at the center of intricate signaling networks, play crucial roles in maintaining health and driving disease progression. SFXN2, a recently identified member of the mitochondrial transporter family, is localized to the outer mitochondrial membrane and regulates several critical mitochondrial functions, including iron metabolism, heme biosynthesis, bioenergetics, and redox homeostasis. New evidence also suggests a connection between SFXN2 and mitochondrial dysfunction related human diseases such as Parkinson’s disease (PD). Despite growing insights into SFXN2’s roles across various mitochondrial functions, its regulation under mitochondrial dysfunction and the resulting biological consequences remains unclear.

**Methods:**

The expression levels of SFXN2 protein were analyzed by Western blotting WB. The interaction between SFXN2 and Parkin was examined using co-immunoprecipitation and immunofluorescence assays. Furthermore, the effect of Parkin on SFXN2 ubiquitination was assessed via ubiquitination assay. Finally, RNA sequencing and flow cytometry were employed to investigate that SFXN2 regulates the apoptotic pathway.

**Results:**

In this study, we identify SFXN2 as a key regulator of mitochondrial homeostasis, demonstrating that its level is tightly regulated via Parkin-mediated ubiquitination and proteasomal degradation. Under conditions of mitochondrial damage, Parkin enhances the degradation of SFXN2, and the reduction of SFXN2 contributes to apoptotic cell death. Functional studies across multiple cell lines, including HEK293, SH-SY5Y, and N2a cells, reveal that the reduction of SFXN2 exacerbates mitochondrial damage-induced apoptosis, whereas overexpression of SFXN2 exhibits an anti apoptotic effect.

**Discussion:**

Our findings offer new insights into the regulation of SFXN2 in mitochondrial dysfunction through Parkin mediated ubiquitin proteasome system activity, underscoring SFXN2’s potential implications in nerodegenerative diseases, particularly PD.

## 1 Introduction

Mitochondria are crucial organelles in eukaryotic cells, not only responsible for generating essential ATP molecules but also serving as centers for synthesizing vital biomolecules such as heme, phospholipids, nucleotides, and amino acids (Kuhlbrandt, 2015). They also play a vital role in regulating apoptosis activity and determining cell fate in response to intrinsic genotoxic stress or extrinsic signals (Wang and Youle, 2009). To facilitate these diverse and complex functions, mitochondria rely on a large number of metabolite carrier proteins, also known as transporters, to transport various solutes into and out of mitochondria (Horten et al., 2020; Pizzagalli et al., 2021). Among these carrier proteins includes the sideroflexins (SFXN, SCL56) family, a group of evolutionarily conserved proteins whose roles as mitochondrial transporters have only recently been discovered (Tifoun et al., 2021).

The SFXN family consists of five homologs (SFXN1–5) in humans and rodents (Tifoun et al., 2022). Initial research progresses in the SFXN proteins were largely centered around SFXN1. SFXN1 regulates iron homeostasis, as its mutation leads to mitochondrial iron accumulation in flexed-tail mouse, zebrafish, and mammalian cells (Fleming et al., 2001; Acoba et al., 2021; Bao et al., 2021). It also functions as a mitochondrial serine transporter essential for one-carbon metabolism (Kory et al., 2018). In mammalian cells, SFXN1 deficiency disrupts mitochondrial respiration by impairing complex III assembly and reducing coenzyme Q1 levels (Acoba et al., 2021). Given the high amino acid similarity among SFXNs (except SFXN4), early discoveries on SFXN1 have shed light on the functions of other SFXN members, revealing their roles in iron homeostasis and one-carbon metabolism (Kory et al., 2018).

Unlike other SFXNs on the inner mitochondrial membrane (IMM), SFXN2 localizes to the outer mitochondrial membrane (OMM) via its transmembrane domains (Mon et al., 2019). Although its substrates remain unidentified, SFXN2-knockout cells show increased mitochondrial iron, decreased heme levels, and reduced activities of heme-dependent enzymes (Mon et al., 2019). GWAS studies suggest a putative link between SFXN2 and Parkinson’s disease (PD), where mitochondrial dysfunction is a key feature (Simon-Sanchez et al., 2009; Li et al., 2021). Elevated SFXN2 correlates with poor clinical outcomes in multiple myeloma and acute myeloid leukemia, potentially due to its regulations of mitochondrial iron metabolism, bioenergetics, autophagy, and redox homeostasis (Chen Y. et al., 2022). All these findings underscore SFXN’s crucial role in mitochondrial functions and human disease.

Mitochondrial proteins and mitochondrial functions are interdependent, with alterations in one affecting the other and contributing to human diseases (Chen et al., 2023). OMM proteins, in particular, are highly sensitive to mitochondrial dysfunction. Mitochondrial dysfunction, caused by genetic or environmental factors, often induces alterations in OMM proteins, which in turn contribute to the pathology (Palmer et al., 2021). Given recent insights into SFXN2’s roles in various mitochondrial functions, its response to mitochondrial dysfunction and the resulting biological consequences remain unexplored.

In this study, we confirm that the protein level of SFXN2 decreases significantly during mitochondrial damage, primarily due to proteasome-mediated degradation. Parkin, a mitochondrial E3 ligase, facilitates the polyubiquitination and proteasomal degradation of SFXN2. Reduced SFXN2 exacerbates mitochondrial damage-induced cell death across multiple cell lines, while elevated expression of SFXN2 protects neurons from the mitochondrial stress. This work identifies SFXN2, as a Parkin substrate, that mitigates mitochondrial damage and enhances cell survival, highlighting its therapeutic potential for neurodegenerative disease like PD.

## 2 Materials and methods

### 2.1 Cell culture

HEK293, SH-SY5Y, and Neuro-2a cells were purchased from Pricella Biotechnology (Wuhan, China), and HeLa cells were provided by Prof. Zhang from University of South China. HEK293 and HeLa cells were cultured in Dulbecco’s modified Eagle’s Medium (DMEM, Cat# C11995500BT, Gibco, USA) with 10% fetal bovine serum (FBS, Cat# F8318, Sigma-Aldrich, USA) and penicillin/streptomycin (Cat# 15140122, Gibco, USA). SH-SY5Y and Neuro-2a cells were maintained in DMEM/Nutrient Mixture F12 (Cat# C11330500BT, Gibco, USA) with the same supplements. All cells were cultured at 37°C in a humidified CO2 incubator.

### 2.2 Plasmids

Plasmids pcDNA3.1-Parkin-GFP, pcDNA3.1-Parkin-HA, pcDNA3.1-Parkin-Myc, pcDNA3.1-Parkin-T240R, and pcDNA3.1-Parkin-R42P were previously described (Xiong et al., 2009). PcDNA3.1-HA-Ub variants (wild type, K48, K48R, K63, K63R, and KR) were generously provided by Prof. Zhang from University of South China. SFXN2-Myc, SFXN2-Flag and SFXN2-HA plasmids were generated by PCR amplification and subcloned into pcDNA3.1 vector. PcSLenti-EF1-Puro-CMV-SFXN2-HA-WPRE and pCLenti-U6-shRNA-CMV-Puro-WPRE containing shRNA targeting SFXN2 were purchased from OBiO Technology (Shanghai, China). The forward targeting sequences of shRNAs are SFXN2 shRNA1 5′–3′: GCTTGAGAAATTGCACTTCAT; SFXN2 shRNA2 5′–3′: GCATCACCCAAGTAGTTATTT; and SFXN2 shRNA3 5′–3′: GAGAGTGAAGCACTTCCTAAA.

### 2.3 Transfection

Transient transfections of plasmid DNA were performed using Lipofectamine 3,000 (Cat# L3000015, Thermo Fisher Scientific, USA) according to the manufacturer’s protocol. SiRNAs were purchased from Genepharma (Shanghai, China) and transfected using Lipofectamine RNAiMAX (Cat# 13778150, Thermo Fisher Scientific, USA) following the manufacturer’s instructions. The sequences of all siRNAs used in this study included hSFXN2 siRNA1 5′–3′: GCGCAUGUCUUUCCAGCUUTT; hSFXN2 siRNA2 5′–3′: GCGGCUAACUGUGUCAAUATT; hSFXN2 siRNA3 5′–3′: GCAUCACCCAAGUAGUUAUTT; Parkin siRNA1 5′–3′: GGAUCAGCAGAGCAUUGUUTT; Parkin siRNA2 5′–3′: GCUCCAUCACUUCAGGAUUTT; Parkin siRNA3 5′–3′: CCUUCUGCCGGGAAUGUAATT; mSFXN2 siRNA1 5′–3′: GCUUCAUGCUGCAGUUCUATT; mSFXN2 siRNA2 5′–3′: GACUACACCUGAUGAAGAATT; and mSFXN2 siRNA3 5′–3′: GGAGAAGAUGAAUGUCAUUTT.

### 2.4 Construction of stable cell lines

HEK293 cells with stable overexpressing or knockdown of SFXN2 were generated by lentiviral transduction. Lentiviral particles carrying control vector, SFXN2-HA, shRNA targeting SFXN2, or scrambled shRNA were individually packaged in HEK293T cells. HEK293T cells were infected with corresponding lentiviruses, and transduced cells were selected over 2 weeks by culturing in medium supplemented with 1.2 μg/mL puromycin (Cat# ST551, Beyotime, China).

### 2.5 Western blot and co-immunoprecipitation

Western blot (WB) was performed as previously described (Li et al., 2023). The antibodies used in WB are listed at the Supplementary Table S3.

For co-immunoprecipitation (co-IP) experiments, cells were scraped off plates using IP lysis buffer (Cat# P0013, Beyotime, China) containing the protease inhibitor cocktail (Cat# S7380, S7381, and S7377, Selleck, USA) and lysed at 4°C for 1 h, followed by centrifugation at 15,000 × g at 4°C for 30 min to remove the debris. Target proteins were immunoprecipitated by incubating the supernatant with appropriate antibodies plus protein A/G-agarose beads (Cat# 20423, Thermo Fisher Scientific, USA), anti-Myc agarose beads (Cat# A7470, Sigma-Aldrich, USA), or anti-HA agarose beads (Cat# 88836, Thermo Fisher Scientific, USA) at 4°C overnight. The beads were washed five times with IP lysis buffer, and the proteins were eluted by heating in 2% SDS buffer for 5 min.

### 2.6 In cell ubiquitination assay

Ubiquitination was analyzed as previously described (Xiong et al., 2009). Briefly, transfected cells were lysed with 2% SDS lysis buffer (2% SDS, 150 mM NaCl, 10 mM Tris–HCl, pH 8.0, 2 mM sodium orthovanadate, 50 mM sodium fluoride, 1 × protease inhibitor cocktail) by boiling at 95°C for 10 min followed by sonication. The lysates were diluted at a 1:9 ratio with dilution buffer (10 mM Tris–HCl, pH 8.0, 150 mM NaCl, 2 mM EDTA, 1% Triton X-100) and incubated on a rotary shaker at 4°C for 1 h, followed by centrifugation at 20,800 ×*g* for 30 min. Subsequently, 1.5 mg of protein was subjected to immunoprecipitation. The immunoprecipitated proteins were washed five times with wash buffer (10 mM Tris–HCl, pH 8.0, 1 M NaCl, 1 mM EDTA, 1% NP-40) and then eluted by boiling in SDS loading buffer at 95°C for 10 min. The eluted proteins were separated by SDS-PAGE. Immunodetection was performed using antibodies against ubiquitin and the precipitated proteins.

### 2.7 *In vitro* ubiquitination assay

*In vitro* ubiquitination assay was performed as described (Xiong et al., 2009). Purified SFXN2-Flag (20 μM) was incubated in ubiquitination assay buffer (50 mM Tris–HCl pH 8.0, 5 mM MgCl_2_, 2 mM DTT) with 100 nM E1 (Cat# E-305–025, Boston Biochem, USA), 1 μM E2 UbcH7 (Cat# E2-640–100, Boston Biochem, USA), 100 μM ubiquitin (Cat# U-100H-10 M, Boston Biochem, USA), and 1 μM recombinant human Parkin pS65 Protein (Cat# E3-166–025, Boston Biochem, USA) at 37°C for 30 min. Reactions were stopped with SDS buffer, immunoprecipitated with anti-Flag agarose beads (Cat# A2220-5ML, Millipore, USA), and analyzed by SDS-PAGE for SFXN2 ubiquitination.

### 2.8 Mitochondria isolation

Mitochondria were purified as described (Li et al., 2023). Cells were harvested, resuspended in Cell Lysis Buffer (10 mM Tris–Hcl, pH 7.4, 250 mM sucrose, 1 mM EDTA, and proteinase inhibitors), and incubated on ice for 15 min. After homogenization with a 2 mL dounce (Cat# F519062-0001, Sangon Biotech, China), lysates were centrifuged at 1,000 g for 10 min at 4°C to remove debris. Mitochondria were then pelleted from the supernatant by centrifugation at 11,000 x g for 10 min.

### 2.9 Immunofluorescence and confocal microscopy

HeLa cells on glass coverslips were fixed with 4% PFA for 10 min, permeabilized with 0.2% Triton X-100 in PBS for 15 min, and blocked in 5% BSA for 1 h. Cells were incubated with primary antibodies at RT for 2 h, washed, then incubated with fluorescent secondary antibodies for another 2 h. Coverslips were mounted with DAPI-containing medium (Cat# ab104139, Abcam, USA) and imaged using an LSM900 Airyscan2 confocal microscope (Zeiss, Germany). The antibodies used in immunofluorescence staining are listed at the Supplementary Table S3.

### 2.10 RNA extraction and real-time quantitative PCR

Total RNA was isolated using TRIzol Reagent (Cat# AM9738, Invitrogen, USA) according to the manufacturer’s instructions. Complementary DNA (cDNA) was obtained using RevertAid First Strand cDNA Synthesis Kit (Cat# K16225, Thermo Fisher Scientific, USA). Quantitative real-time PCR was performed using Maxima SYBR Green qPCR Solution (Cat# K0252, Thermo Fischer Scientific, USA) on the QuantStudio 5 Real-Time PCR (Thermo Fisher Scientific, USA). The sequences of primers used are listed at the Supplementary Table S4. The CT values of ACTIN were used for normalization, and the 2^−ΔΔCt^ method was used to calculate the relative expression levels of the target gene (Livak and Schmittgen, 2001).

### 2.11 Flow cytometry of apoptosis

Cells were stained using the FITC Annexin V Apoptosis Detection Kit (Cat# 556547, BD Biosciences, USA), which includes both annexin V-fluorescein isothiocyanate (FITC) and propidium iodide (PI). After incubation for 15 min at 25°C in the dark, the ratio of apoptotic cell (early apoptosis: annexin V-FITC^+^/PI^−^; late apoptosis: annexin V-FITC^+^/PI^+^) was analyzed in triplicates using a CytoFLEX SRT Cell Sorter (Beckman Coulter Inc., USA). Data were analyzed using FlowJo v.10.4 software (Ashland, USA).

### 2.12 Statistical analyses

Data are represented as mean ± SEM. Unpaired two-tailed Student’s *t*-test was used to compare means between two groups; one-way ANOVA was used to compare means among three or more groups for one categorical independent variable; two-way ANOVA was used to compare means among three or more groups for two categorical independent variables. *p* values less than 0.05 were considered statistically significant: *p < 0.05, **p < 0.01, ***p < 0.001, ****p < 0.0001, ns, non-significant. *p* values greater than 0.05 were considered not statistically significant.

## 3 Results

### 3.1 Proteasome-mediated degradation of SFXN2 during mitochondrial damage

**Figure 1:**
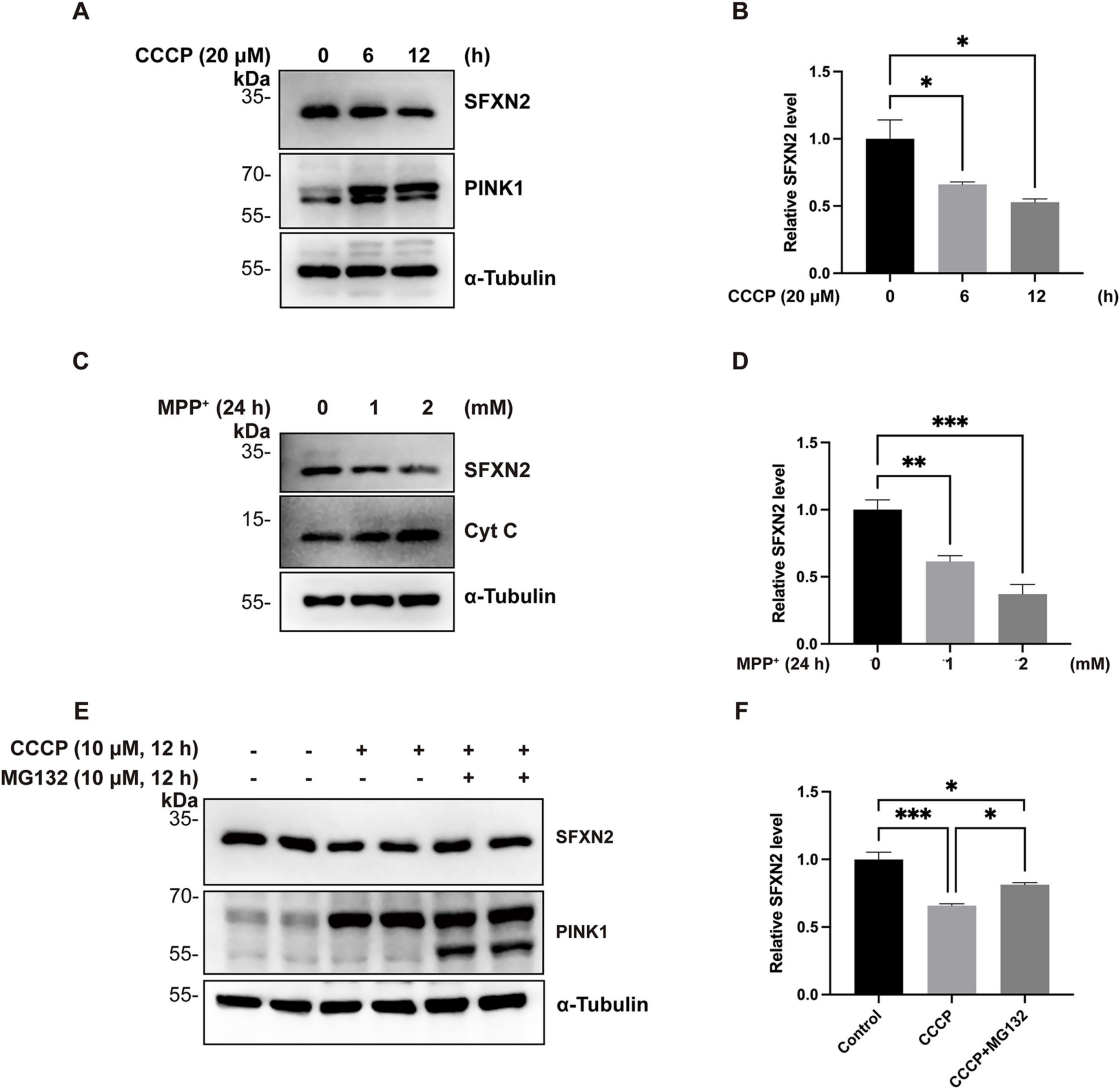
Mitochondrial impairment decreases SFXN2 levels **(A,B)** Western blot (WB) analysis of SFXN2 and PINK1 protein levels in HEK293 cells treated with 20 μM CCCP for the indicated time periods **(A)**. Quantification of relative SFXN2 protein levels normalized to *α*-Tubulin is presented in the histogram **(B)**. **(C,D)** WB analysis of SFXN2 and Cytochrome C (Cyt C) protein levels in HEK293 cells treated with the indicated concentrations of MPP^+^ for 24 h **(C)**. Quantification of relative SFXN2 protein levels normalized to α-Tubulin is presented in the histogram **(D)**. **(E,F)** WB analysis of SFXN2 and PINK1 protein levels in HEK293 cells treated with vehicle control (0.05% (v/v) DMSO), CCCP (10 μM) alone, or CCCP (10 μM) combined with MG132 (10 μM) for 12 h **(E)**. Quantification of relative SFXN2 protein levels normalized to actin is presented in the histogram **(F)**. Images are representative of at least three independent experiments with similar results. Histogram data are presented as mean ± SEM (*N* = 3). Statistical significance was analyzed using one-way ANOVA. *p < 0.05, **p < 0.01, ***p < 0.001.

Mitochondrial proteins are highly sensitive to functional and membrane potential changes in mitochondria (Zorova et al., 2018). To examine the behaviour of SFXN2 under mitochondrial stress, HEK293 cells were treated with carbonyl cyanide m-chlorophenylhydrazone (CCCP), a mitochondria uncoupler that disrupts the proton gradient across IMM. As expected, prolonged CCCP exposure led to time- and dose-dependent accumulation of full-length PINK1 (~62 kDa), a hallmark of mitochondrial depolarization, confirming an effective induction of mitochondrial dysfunction (Matsuda et al., 2010). In parallel, SFXN2 protein levels progressively declined under the same conditions (Figures 1A,B; Supplementary Figures S1A,B).

To further assess the generality of this phenomenon, additional mitochondrial toxins, including 1-methyl-4-phenylpyridinium (MPP^+^, a mitochondrial complex I inhibitor), carbonyl cyanide-p-trifluoromethoxyphenylhydrazone (FCCP, another mitochondrial uncoupler), oligomycin A (an mitochondrial ATP synthase inhibitor), and rotenone (another complex I inhibitor) were tested (Heytler and Prichard, 1962; Ramsay et al., 1989; Heinz et al., 2017; Yang et al., 2021; Mackieh et al., 2023). Treatment with each of these agents similarly led to reduced SFXN2 protein levels (Figures 1C,D; Supplementary Figures S1C–G), suggesting that SFXN2 downregulation is a common response to mitochondrial dysfunction.

Given SFXN2’s OMM localization (Mon et al., 2019), we extracted mitochondria from HEK293 cells and found that SFXN2 levels were reduced in both whole-cell extracts and mitochondrial fractions upon CCCP treatment, with no detectable cytoplasmic SFXN2 (Supplementary Figure S1H). These findings confirms that SFNX2 remains mitochondrial-associated and is decreased upon mitochondrial damage.

Considering the well-established role of the ubiquitin-proteasome system (UPS) in mitochondrial protein homeostasis, we hypothesized the observed SFXN2 reduction upon mitochondrial damage may be UPS-dependent (Livnat-Levanon and Glickman, 2011). To test this hypothesis, HEK293 cells were treated with CCCP in the presence or absence of proteasome inhibitor MG132. As expected, CCCP treatment for 12 h led to a substantial decrease in endogenous SFXN2 protein levels. Co-treatment with MG132 significantly attenuated this reduction, restoring SFXN2 levels by 55.6% ± 8% compared to the CCCP-only group (Figures 1E,F; Supplementary Figures S1I–J). Therefore, mitochondrial damage triggers proteasome-mediated degradation SFXN2.

### 3.2 Parkin negatively regulates SFXN2 level via UPS

**Figure 2:**
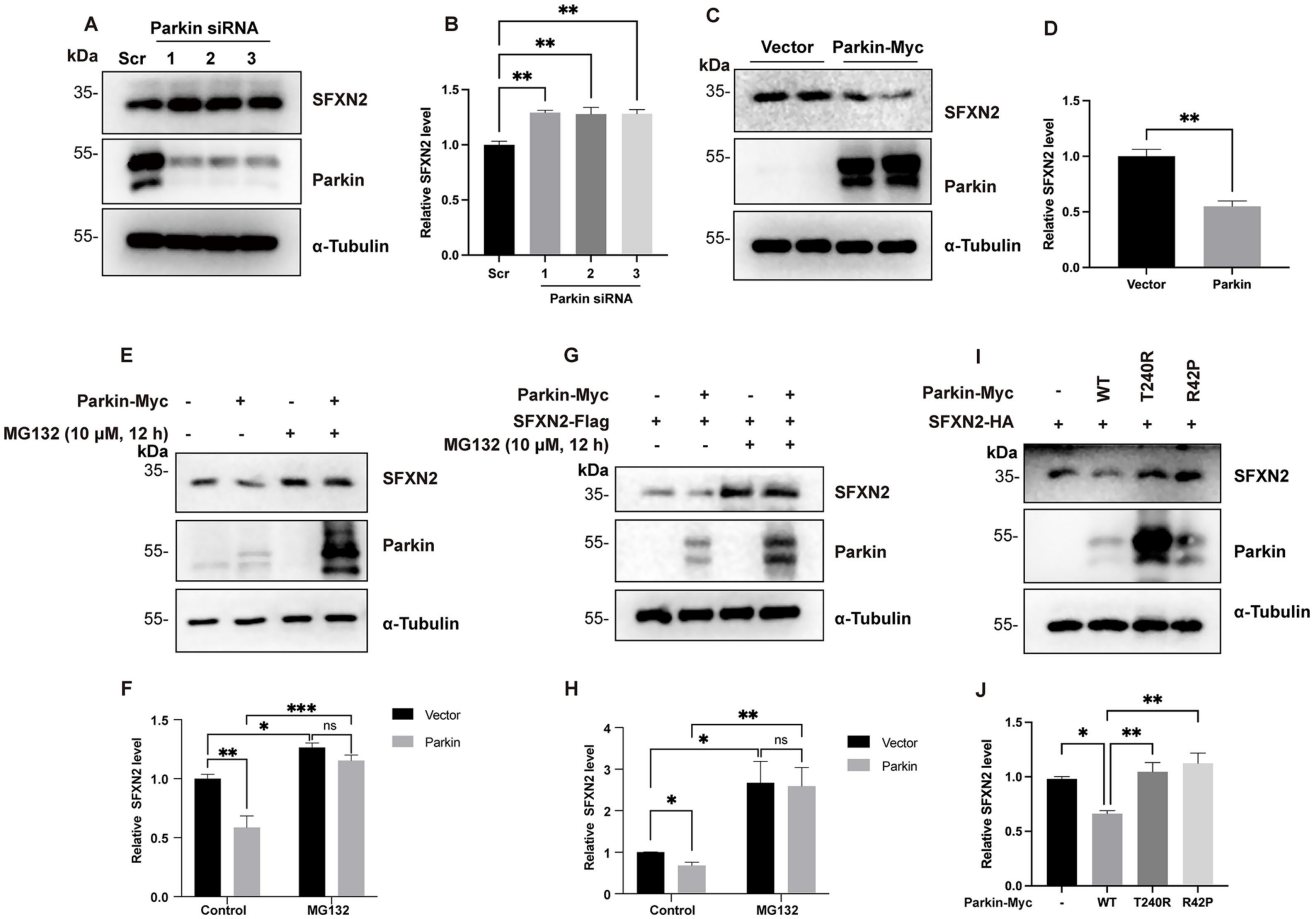
Parkin negatively regulates SFXN2 protein levels in cells **(A,B)** Western blot (WB) analysis of SFXN2 and Parkin protein levels in HEK293 cells transfected with scrambled siRNA or siRNA targeting Parkin **(A)**. Quantification of relative SFXN2 protein levels normalized to α-Tubulin is presented in the histogram **(B)**. **(C,D)** WB analysis of SFXN2 and Parkin protein levels in HEK293 cells overexpressing Parkin-Myc or control vector **(C)**. Quantification of relative SFXN2 protein levels normalized to α-Tubulin is presented in the histogram **(D)**. **(E,F)** WB analysis of endogenous SFXN2 and Parkin protein levels in HEK293 cells overexpressing control vector or Parkin-Myc **(E)**, with or without 10 μM MG132 treatment for 8 h before harvest. Quantification of relative SFXN2 protein levels normalized to α-Tubulin is presented in the histogram **(F)**. **(G,H)** WB analysis of SFXN2 and Parkin protein levels in HEK293 cells overexpressing SFXN2-HA alone or Parkin-Myc plus SFXN2-HA **(G)**, with or without 10 μM MG132 treatment for 8 h before harvest. Quantification of relative SFXN2 protein levels normalized to α-Tubulin is presented in the histogram **(H)**. **(I,J)** WB analysis of SFXN2 and Parkin protein levels in HEK293 cells expressing SFXN2-HA and Parkin variant (control vector, Parkin-WT, Pakin-T240R, or Parkin-R42P). Anti-α-Tubulin was used as the loading control for the IB. Quantification of relative SFXN2 protein levels normalized to α-Tubulin is presented in the histogram **(J)**. Images are representative of at least three independent experiments that gave similar results. Histogram data are presented as mean ± SEM (*N* = 3). Statistical significance was analyzed using one-way ANOVA and two-way ANOVA. *p < 0.05, **p < 0.01, ***p < 0.001, ns, non-significant.

Parkin is a well-characterized E3 ubiquitin ligase that targets a wide range of OMM proteins on damaged mitochondria for proteasomal degradation. Given its established role in mitochondrial protein turnover, we hypothesized that Parkin might mediate SFXN2 degradation via UPS. To investigate this possibility, we manipulated Parkin expression and observed the effects on SFXN2 levels. In HEK293 cells, SFXN2 levels significantly increased with Parkin knockdown and decreased upon Parkin overexpression (Figures 2A–D). MG132 co-treatment effectively blocked Parkin overexpression-induced reduction of endogenous SFXN2 (Figures 2E,F) or exogenously expressed SFXN2 (Figures 2G,H), suggesting Parkin promotes SFXN2 degradation through a proteasome-dependent mechanism.

To assess the requirement of Parkin’s E3 ligase activity in promoting SFXN2 degradation, we examined the effects of two inactive Parkin mutants (T240R and R42P) on SFXN2 levels (Seirafi et al., 2015). Unlike wild-type Parkin, neither mutant significantly altered SFXN2 levels (Figures 2I–J), demonstrating that Parkin’s E3 ligase activity is essential for UPS-mediated SFXN2 degradation. Together, these results suggest that SFXN2 is a novel Parkin substrate.

### 3.3 Parkin interacts with SFXN2 on mitochondria

**Figure 3:**
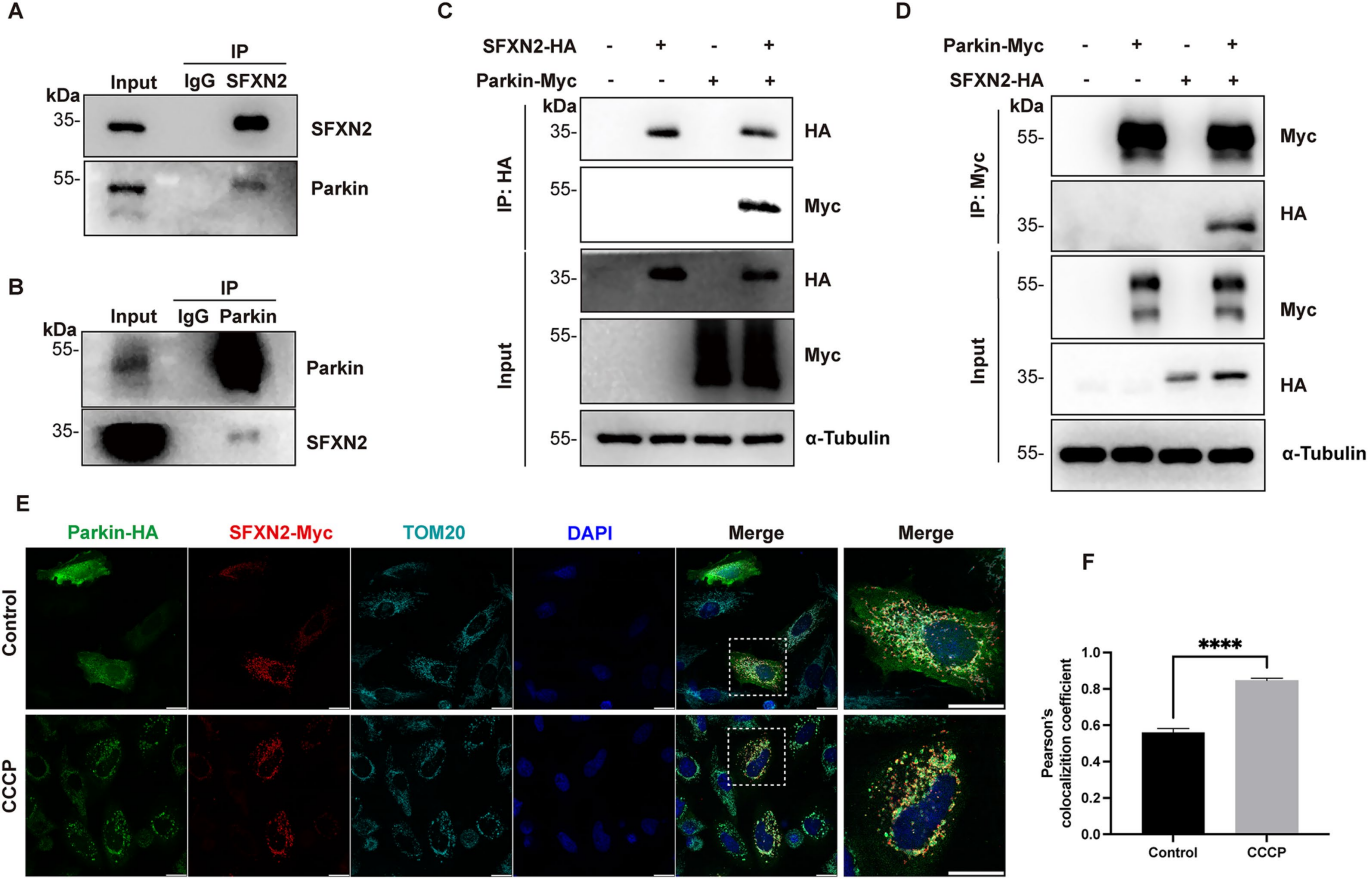
Parkin interacts with SFXN2 **(A,B)** Western blot (WB) analyses of the co-immunoprecipitation (co-IP) of endogenous Parkin and SFXN2 in HEK293 cells. Lysates of HEK293 were immunoprecipitated with unspecific IgG **(A,B)**, anti-SFXN2 **(A)**, or anti-Parkin **(B)** antibody, followed by immunobloting (IB) with anti-SFXN2 and anti-Parkin antibodies. Input proteins were used as controls for the IB. **(C,D)** WB analyses of the co-IP of Parkin-HA with SFXN2-Myc **(C)** and the co-IP of SFXN2-HA with Parkin-Myc **(D)** in transiently transfected HEK293 cells expressing the indicated constructs or control vector. Lysates of the transfected HEK293 were immunoprecipitated with anti-Myc antibody, followed by IB with anti-Myc and anti-HA antibodies **(C,D)**. Input proteins were used as controls for the IB. **(E,F)** Immunofluorescent staining of HeLa cells overexpressing Parkin-HA and SFXN2-Myc, with the addition of 20 μM CCCP or vehicle control (0.05% (v/v) DMSO) for 6 h. Cells were immunostained with anti-HA (green), anti-Myc (red), and anti-TOM20 (light blue) antibodies. Scale bars, 20 μm. The histogram **(F)** presents the quantification of Pearson’s correlation coefficient between Parkin-HA and SFXN2-Myc. Images are representative of at least three independent experiments that gave similar results. Histogram data are presented as mean ± SEM (*N* = 3). Statistical significance was analyzed using Student’s *t*-test. ****p < 0.0001.

E3 ubiquitin ligases typically bind directly to their substrates to ensure substrate specificity in UPS degradation. To explore this relation between Parkin and SFXN2, we carried out co-IP experiments. In HEK293 cell lysates, endogenous Parkin and SFXN2 reciprocally co-precipitate, demonstrating their interaction under basal conditions (Figures 3A,B), and this interaction was further validated between exogenously expressed Parkin and SFXN2 (Figures 3C,D). In contrast, SFNX2 does not interact with BAX, a well-characterized Parkin substrate (Johnson et al., 2012), supporting a direct interaction between Parkin and SFXN2 (Supplementary Figure S2A).

To further characterize the subcellular context of this interaction, we first performed immunofluorescence analysis on HeLa cells (which are bigger cells thus giving better subcellular images than HEK293 cells). Under basal conditions, overexpressed Parkin displayed a diffused cytosolic distribution, whereas SFXN2 colocalized with the mitochondrial marker TOM20, consistent with it OMM localization. Upon CCCP-induced mitochondrial depolarization, Parkin relocalized to TOM20-labelled mitochondria as previous reported (Narendra et al., 2010), and the overlap between Parkin and SFXN2 signals significantly increased (Figures 3E,F). A similar enhancement in colocalization was also observed in HEK293 cells under similar conditions (Supplementary Figures S2B,C). Together, these results demonstrate that Parkin interacts with SFXN2 on mitochondria, and mitochondrial depolarization enhances their interaction.

### 3.4 Parkin polyubiquitinates SFXN2 *in vitro* and in cells

**Figure 4:**
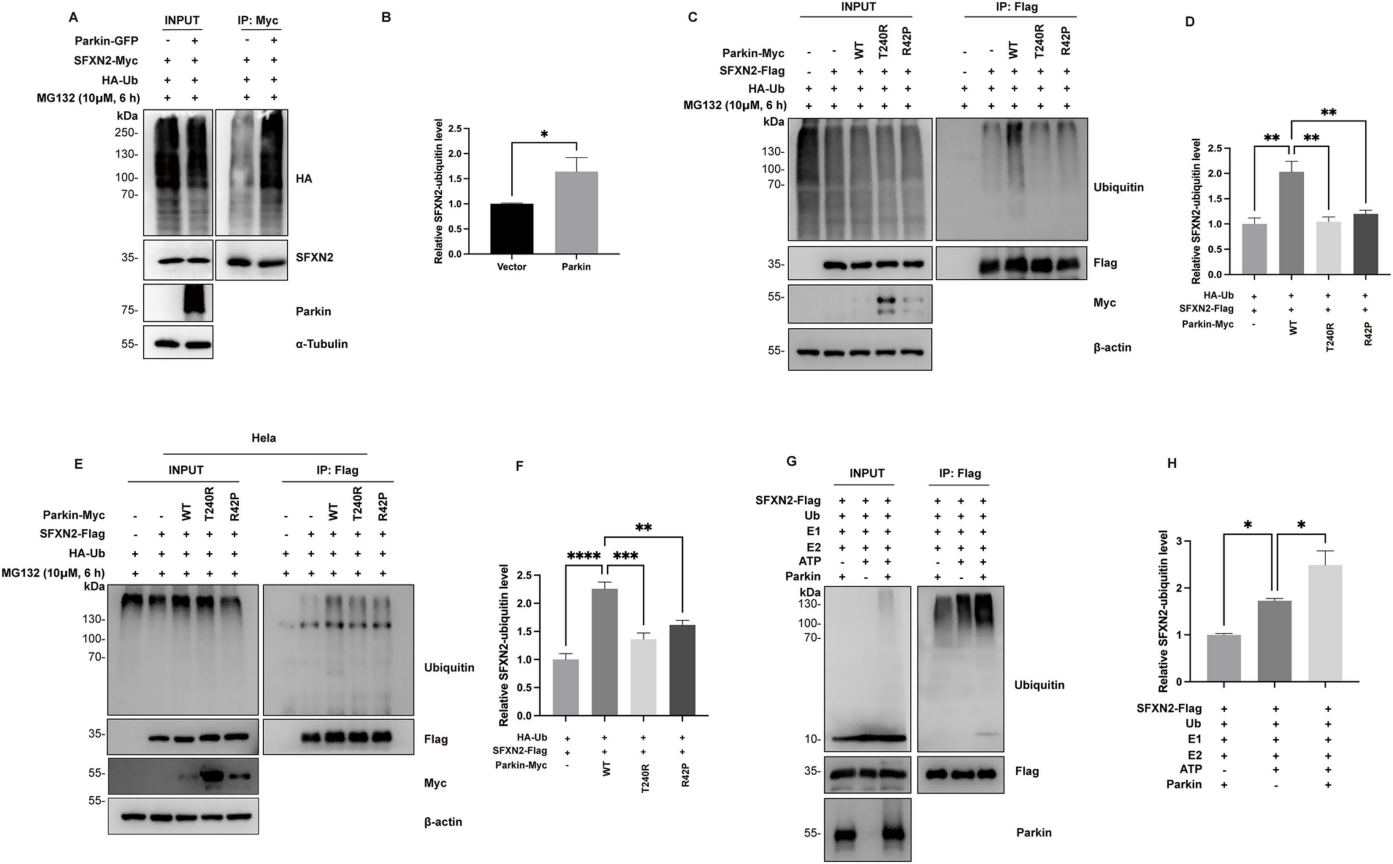
Parkin polyubiquitinates SFXN2 in cells and *in vitro* **(A,B)** Western blot (WB) analysis of ubiquitination of SFXN2-Myc in transiently transfected HEK293 cells expressing SFXN2-Myc and HA-Ubiquitin (HA-Ub), with or without Parkin-GFP. Cells were treated with 10 μM MG132 for 6 h before harvest. SFXN2-Myc in cell lysates was immunoprecipitated with anti-Myc antibody, followed by immunoblotting (IB) with anti-HA and anti-SFXN2 antibodies. Input proteins were immunoblotted with anti-HA, anti-SFXN2, anti-Parkin, and anti-α-Tubulin as controls for the IB **(A)**. Quantification of relative SFXN2 polyubiquitination levels normalized to SFXN2 is presented in the histogram **(B)**. **(C,D)** WB analysis of ubiquitination of SFXN2-Flag in transiently transfected HEK293 cells expressing SFXN2-Flag and HA-Ub, together with Parkin variant (control vector, Parkin-WT, Pakin-T240R, or Parkin-R42P). Cells were treated with 10 μM MG132 for 6 h before harvest. SFXN2-Flag in cell lysates was immunoprecipitated with anti-Flag antibody, followed by IB with anti-Ubiquitin. Input proteins were immunoblotted with anti-Ubiquitin, anti-Flag, anti-Myc, and anti-α-Tubulin or *β*-actin as controls for the IB **(C)**. Quantification of relative SFXN2 polyubiquitination levels normalized to SFXN2-Flag is presented in the histogram **(D)**. **(E,F)** WB analysis of ubiquitination of SFXN2-Flag in transiently transfected Hela cells expressing SFXN2-Flag and Parkin variant (control vector, Parkin-WT, Pakin-T240R, or Parkin-R42P). Cells were treated with 10 μM MG132 for 6 h before harvest. SFXN2-Flag in cell lysates were immunoprecipitated with anti-Flag antibody, followed by IB with anti-Ubiquitin. Input proteins were immunoblotted with anti-Ubiquitin, anti-Flag, anti-Myc, and anti-α-Tubulin or β-actin as controls for the IB **(E)**. Quantification of relative SFXN2 polyubiquitination levels normalized to SFXN2-Flag is presented in the histogram **(F)**. **(G,H)** WB analysis of *in vitro* ubiquitination of SFXN2-Flag using recombinant E1 (UBE1), recombinant E2 (UbcH7), Ubiquitin, and Parkin. The reaction products were immunoblotted with anti-Ubiquitin, anti-SFXN2, and anti-Parkin antibodies **(G)**. Histogram presenting the quantification of relative SFXN2 polyubiquitination levels normalized to SFXN2-Flag, in the presence or absence of Parkin **(H)**. Images are representative of at least three independent experiments with similar results. Histogram data are presented as mean ± SEM (*N* = 3). Statistical significance was analyzed using one-way ANOVA.*p < 0.05, **p < 0.01, ***p < 0.001, ****p < 0.0001.

To assess whether Parkin mediates SFXN2 ubiquitination, HEK293 cells co-expressing SFXN2 and ubiquitin (Ub), with or without Parkin, were analyzed. Our results showed that Parkin expression significantly increased SFXN2 polyubiquitination (Figures 4A,B). To assess the role of Parkin’s E3 ligase activity in this process, we examined the effects of Parkin variants on SFXN2 polyubiquitination. As shown in Figure 4C, after treating cells with MG132 to block protein degradation and preserve SFXN2 ubiquitination, only wild-type Parkin, but not loss-of-function T240R and R42P Parkin mutants, promoted SFXN2 polyubiquitination (Figures 4C,D). Similar results were observed in HeLa cells lacking endogenous parkin, where wild-type Parkin increased SFXN2 polyubiquitination, but T240R and R42P Parkin mutants did not (Figures 4E,F). Notably, SFXN2 polyubiquitination still occurred in the absence of Parkin, suggesting other E3 ligases may also be able to target SFXN2 (Figure 4E).

To further confirm Parkin’s role in SFXN2 polyubiquitination, we conducted *in vitro* ubiquitination assays using precipitated SFXN2 from HEK293 cells and purified Parkin pS65 (a constitutively active Parkin mutant). Endogenous level of SFXN2 ubiquitination occurred without adding additional Parkin and with the presence of E1 ligase ubiquitin-activating enzyme E1 (UBE1), E2 ligase ubiquitin-conjugating enzyme H7 (UbcH7) and Ub. However, ubiquitinated SFXN2 bands markedly increased upon the addition of Parkin pS65, confirming that Parkin directly facilitates SFXN2 polyubiquitination as an E3 ligase (Figures 4G,H).

**Figure 5:**
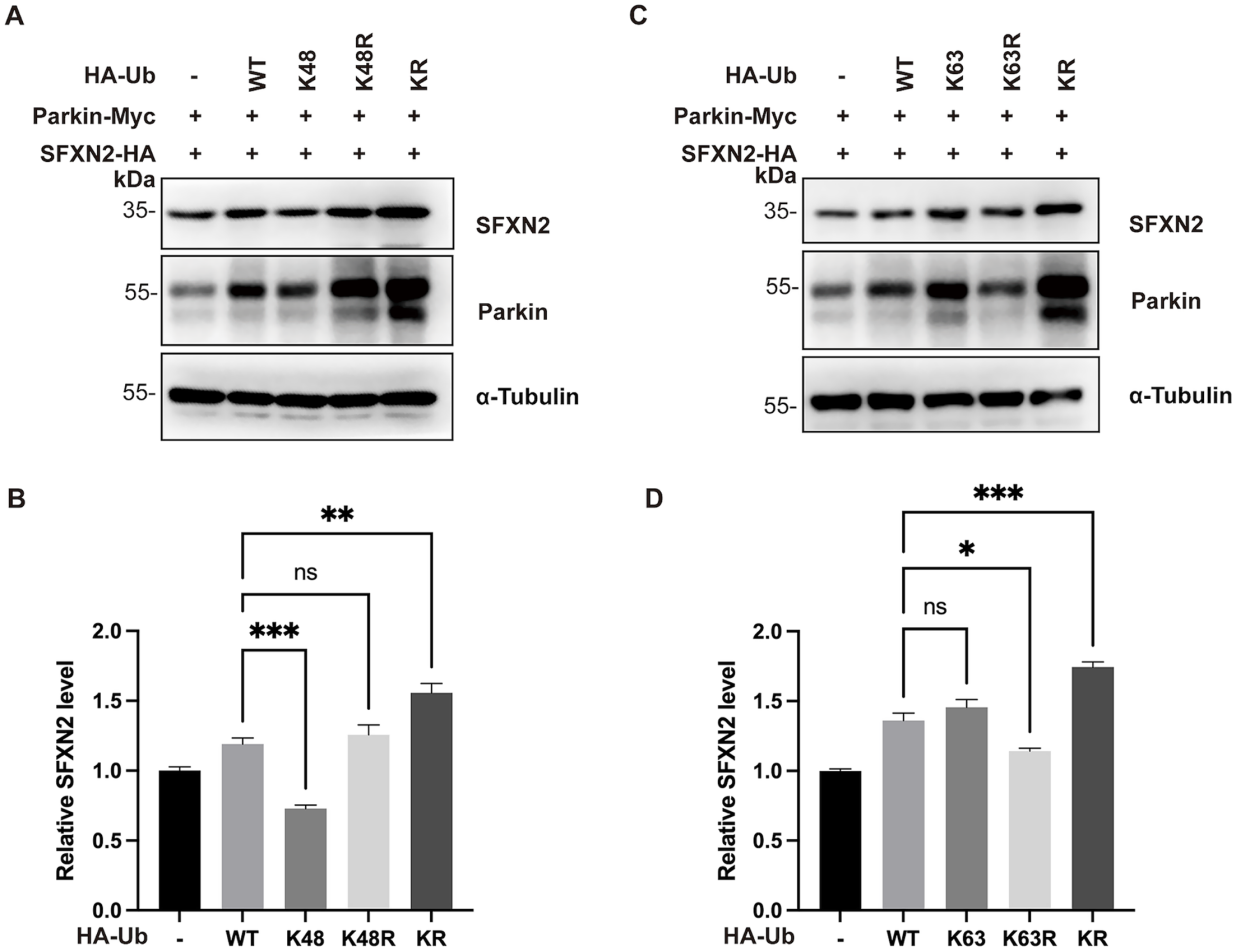
Parkin mediates SFXN2 degradation through K48-linked polyubiquitination **(A,B)** Western blot (WB) analysis of SFXN2, Parkin, and Ubiquitin protein levels in HEK293 cells expressing Parkin-Myc, SFXN2-HA, and Ubiquitin variant (control vector, HA-Ub-WT, HA-Ub-K48, HA-Ub-K48R, or HA-Ub-KR). Anti-α-Tubulin was used as the loading control for the immunoblotting (IB, **A**). Quantification of relative SFXN2 protein levels normalized to α-Tubulin is presented in the histogram **(B)**. **(C,D)** WB analysis of SFXN2, Parkin, and Ubiquitin protein levels in HEK293 cells expressing Parkin-Myc, SFXN2-HA, and Ubiquitin variant (control vector, HA-Ub-WT, HA-Ub-K63, HA-Ub-K63R, or HA-Ub-KR). Anti-α-Tubulin was used as the loading control for the IB **(C)**, Quantification of relative SFXN2 protein levels normalized to α-Tubulin is presented in the histogram **(D)**. Images are representative of at least three independent experiments that gave similar results. Histogram data are presented as mean ± SEM (*N* = 3). Statistical significance was analyzed using one way ANOVA. *p < 0.05, **p < 0.01, ***p < 0.001, ns, non-significant.

Parkin is known to catalyze both canonical K48-linked polyubiquitination, which targets protein for proteasomal degradation, and non-canonical K63-linked polyubiquitination, associated with autophagy activation and other signaling (Seibenhener et al., 2004; Olzmann et al., 2007; Manohar et al., 2019). We thus hypothesized that Parkin mediates SFXN2 degradation through K48-linked ubiquitination and analyzed the effect of ubiquitin mutants on SFXN2 level in HEK293 cells (Nucifora et al., 2016). Ub-K48 and Ub-K63 have arginine substitutions of all lysine residues except K48 and K63, only allowing K48- and K63-linked polyubiquitin chains, respectively; Ub-K48R and Ub-K63R have lysine-to-arginine mutation at K48 and K63, preventing the formation of K48- and K63-linked polyubiquitin chains, respectively; and Ub-KR has all lysine residues substituted with arginine, preventing polyubiquitin chain formation through any lysine. As shown in Figures 5A,B, compared to wild-type Ub (Ub-WT), Ub-K48, but not Ub-K63, significantly reduced SFXN2 levels, while Ub-KR largely increased SFXN2 levels (Figures 5C,D). These results indicate that Parkin primarily mediates SFXN2 degradation via K48-linked ubiquitination.

### 3.5 Parkin facilitates proteasome degradation of SFXN2 during mitochondrial damage

**Figure 6:**
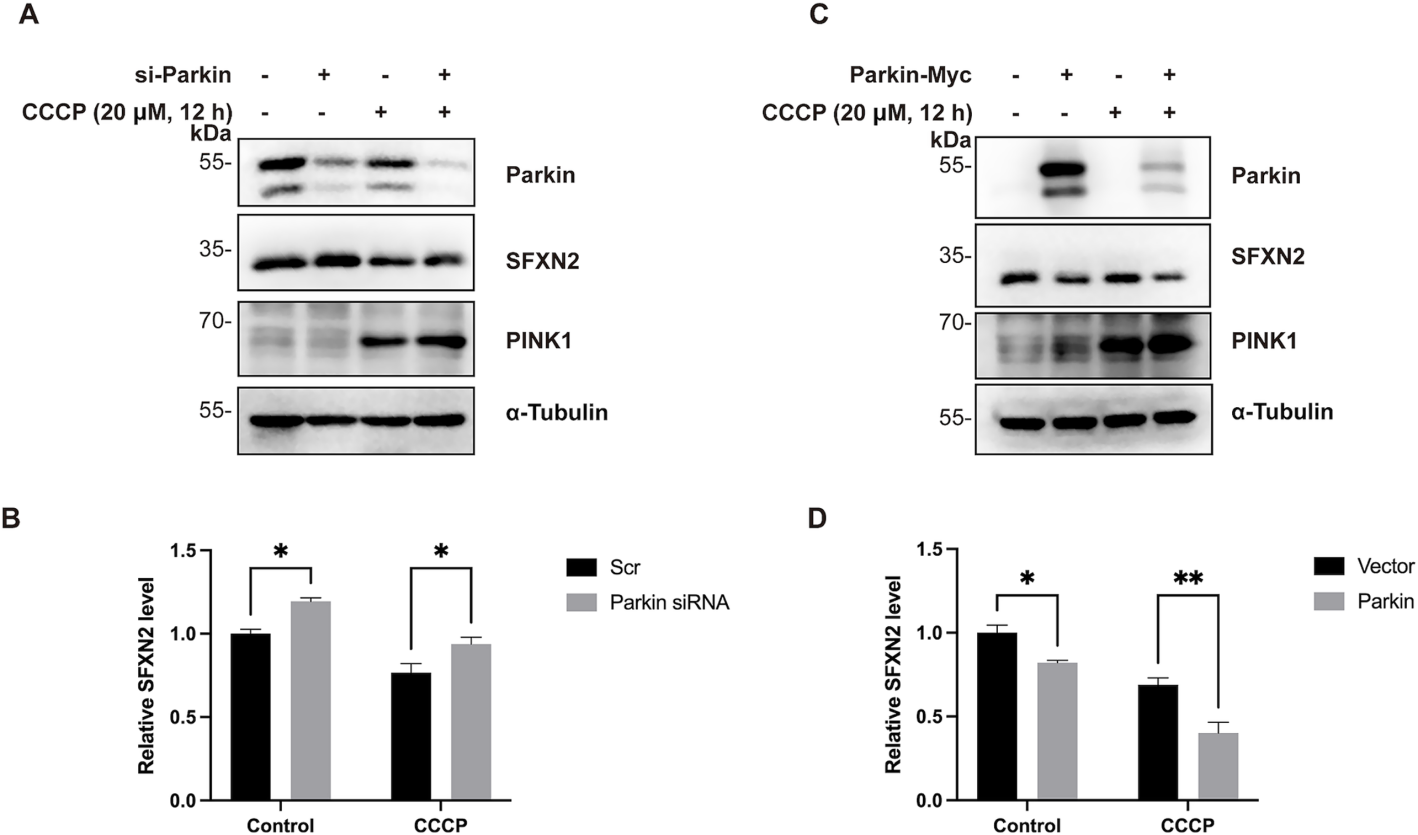
Parkin mediates SFXN2 degradation during CCCP-induced mitochondria damage **(A,B)** Western blot (WB) analysis of SFXN2 and Parkin protein levels in HEK293 cells transfected with scrambled siRNA or siRNA targeting Parkin, with the addition of 20 μM CCCP or vehicle control (0.05% (v/v) DMSO) for 12 h **(A)**. Quantification of relative SFXN2 protein levels normalized to α-Tubulin is presented in the histogram **(B)**. **(C,D)** WB analysis of SFXN2 and Parkin protein levels in HEK293 cells overexpressing Parkin-Myc or a control vector, with the addition of 20 μM CCCP or vehicle control (0.05% (v/v) DMSO) for 12 h **(C)**. Quantification of relative SFXN2 protein levels normalized to α-Tubulin is presented in the histogram **(D)**. Images are representative of at least three independent experiments with similar results. Histogram data are presented as mean ± SEM (*N* = 3). Statistical significance was analyzed using two-way ANOVA. *p < 0.05, **p < 0.01.

Since Parkin is activated upon recruitment to damaged mitochondria, we next investigated whether Parkin mediates the proteasomal degradation of SFXN2 during mitochondrial damage. CCCP treatment induced mitochondrial depolarization, indicated by PINK1 accumulation, and led to a reduction in SFXN2 levels. Consistently, Parkin knockdown alone increased SFXN2 levels, whereas Parkin overexpression alone decreased SFXN2 levels. Notably, Parkin knockdown significantly blocked the CCCP-induced reduction in SFXN2 levels, while Parkin overexpression further enhanced this reduction (Figures 6A–D). These data suggest that Parkin facilitates proteasome degradation of SFXN2 during mitochondrial damage.

### 3.6 SFXN2 protects HEK293 cells against mitochondrial damage-induced apoptosis

**Figure 7:**
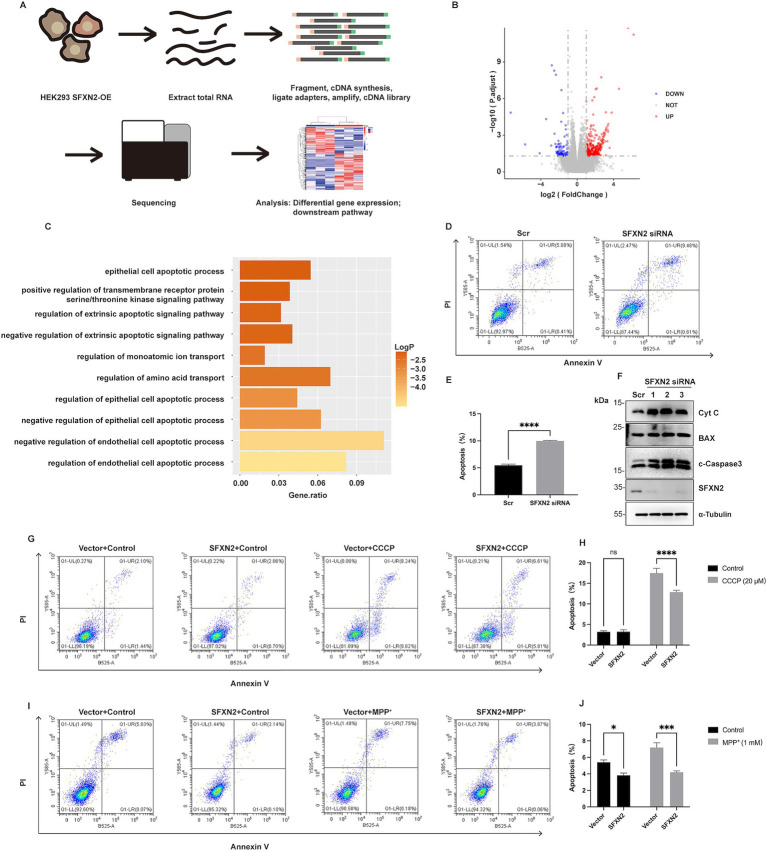
SFXN2 regulates apoptosis in HEK293 cells **(A)** Schematic diagram of RNA sequencing (RNA-seq) experimental design and procedures. **(B)** Volcano plot of differentially expressed genes between SFXN2 overexpressed and control groups (*n* = 3 in each group). Significantly upregulated genes are marked with red dots, and significantly downregulated genes are shown in blue. The horizontal dotted line indicates a *p* value threshold of 0.05, while vertical dotted lines demarcate a fold change of ±2 (Log_2_FC = ±1). **(C)** Bar plot of the top 10 pathways significantly enriched in HEK293 cells overexpressing SFXN2 compared with control cells. **(D,E)** Annexin V/PI staining and flow cytometry assays of HEK293 cells transfected with scrambled siRNA or siRNA targeting SFXN2 **(D)**, with quantification of the percentage of apoptosis cells (Annexin V-positive cells) presented in the histogram **(E)**. **(F)** Western blot analysis of cytochrome C (Cyt C), BAX, cleaved-Caspase3 (c-Caspase3), and SFXN2 protein levels in HEK293 cells transfected with scrambled siRNA or siRNA targeting SFXN2. **(G,H)** Annexin V/PI staining and flow cytometry assays of transiently transfected HEK293 cells expressing SFXN2 or control vector, treated with vehicle control (0.05% (v/v) DMSO) or 20 μM CCCP for 12 h **(G)**, with quantification of the percentage of apoptosis cells (Annexin V-positive cells) presented in the histogram **(H)**. **(I,J)** Annexin V/PI staining and flow cytometry assays of transiently transfected HEK293 cells expressing SFXN2 or control vector, treated with vehicle control (dilution water) or 1 mM MPP^+^ for 24 h **(I)**, with quantification of the percentage of apoptosis cells (Annexin V-positive cells) presented in the histogram **(J)**. Images are representative of at least three independent experiments that gave similar results. Histogram data are presented as mean ± SEM (*N* = 3). Statistical significance was analyzed using two-way ANOVA. *p < 0.05, ***p < 0.001,****p < 0.0001, ns, non-significant.

Given the largely uncharacterized biological function of SFXN2, we next examined the functional consequence of altered SFXN2 levels. RNA sequencing (RNA-seq) of HEK293 cells overexpressing SFXN2 identified 446 differentially expressed genes (DEGs, 361 upregulated and 85 downregulated) (Figures 7A,B; Supplementary Table S1). GO analysis revealed enrichment in known functions of SFXN family proteins, including amino acid transport, ion transport, and transmembrane receptor serine/threonine kinase signalling pathway. Notably, pathways related to negative regulation of epithelial cell apoptotic process were among the most enriched, suggesting a potential anti-apoptotic role for SFXN2 (Figure 7C).

To investigate whether SFXN2 affects apoptosis, we performed double-staining with PI and Annexin V and analyzed the apoptotic population using flow cytometry in HEK293 cells. Results showed that SFXN2 knockdown significantly increased apoptosis, along with elevated cytochrome c, BAX, and cleaved caspase-3 levels (Figures 7D–F). Conversely, SFXN2 overexpression reduced the percentage of apoptotic cells and decreased cleaved-caspase3 levels (Supplementary Figure S3A). Examining the effects of SFXN2 on mitochondrial damage-induced apoptosis, we found that SFXN2 overexpression significantly attenuated apoptosis induced by CCCP and MPP^+^ (Figures 7G–J; Supplementary Figures S3B,C), confirming its anti-apoptotic function. Together, these findings suggest that reduced SFXN2 may contribute to apoptosis resulting from mitochondrial dysfunction.

### 3.7 SFXN2 protects neuronal cells against mitochondrial damage-induced apoptosis

Mitochondrial dysfunction-induced apoptosis is a key neurological pathology in neurodegenerative diseases, including PD. A previous genome-wide association study (GWAS) identified an association between SFXN2 and PD (Simon-Sanchez et al., 2009), prompting us to investigate whether mitochondrial damage affects SFXN2 protein levels in neuronal cells and whether such changes influence neuronal apoptosis.

**Figure 8:**
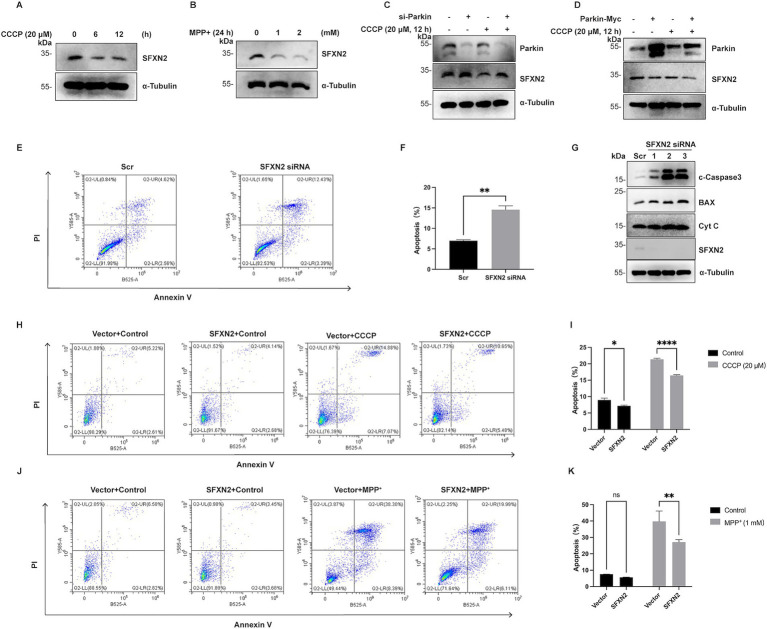
SFXN2 regulates apoptosis in SH-SY5Y **(A,B)** Western blot (WB) analysis of SFXN2 protein levels in SH-SY5Y cells treated with 20 μM CCCP **(A)** for the indicated time periods or with the indicated concentrations of MPP^+^ for 24 h **(B)**. **(C)** WB analysis of SFXN2 and Parkin protein levels in SH-SY5Y cells transfected with scrambled siRNA or siRNA targeting Parkin, followed by treatment with 20 μM CCCP or vehicle control (0.05% (v/v) DMSO) for 12 h. **(D)** WB analysis of SFXN2 and Parkin protein levels in SH-SY5Y cells overexpressing Parkin-Myc or control vector, followed by treatment with 20 μM CCCP or vehicle control (0.05% (v/v) DMSO) for 12 h. **(E,F)** Annexin V/PI staining and flow cytometry assays of SH-SY5Y cells transfected with scrambled siRNA or siRNA targeting SFXN2 **(E)**, with the quantification of the percentage of apoptosis cells (Annexin V-positive cells) presented in the histogram **(F)**. **(G)** WB analysis of cytochrome C (Cyt C), BAX, cleaved-Caspase3 (c-Caspase3), and SFXN2 protein levels in SH-SY5Y cells transfected with scrambled siRNA or siRNA targeting SFXN2. **(H,I)** Annexin V/PI staining and flow cytometry assays of transiently transfected SH-SY5Y cells expressing SFXN2 or control vector, followed by treatment with vehicle control (0.05% (v/v) DMSO) or 20 μM CCCP for 12 h **(H)**. Quantification of the percentage of apoptotic cells (Annexin V-positive cells) is presented in the histogram **(I)**. **(J,K)** Annexin V/PI staining and flow cytometry assays of transiently transfected SH-SY5Y cells expressing SFXN2 or control vector, followed by treatment with vehicle control (0.05% (v/v) DMSO) or 1 mM MPP^+^ for 24 h **(J)**. Quantification of the percentage of apoptotic cells (Annexin V-positive cells) is presented in the histogram **(K)**. Images are representative of at least three independent experiments that gave similar results. Histogram data are presented as mean ± SEM (*N* = 3). Statistical significance was analyzed using two-way ANOVA. *p < 0.05, **p < 0.01, ****p < 0.0001, ns, non-significant.

Mass spectrometry analysis confirmed SFXN2 expression in human and mouse brains, including forebrain, midbrain, and cerebellum (Supplementary Table S2). In SH-SY5Y neuroblastoma cells, both CCCP and MPP^+^ treatment caused a significant SFXN2 reduction (Figures 8A,B), mirroring findings in HEK293 cells. Parkin knockdown increased SFXN2 levels, whereas Parkin overexpression reduced them. Under CCCP treatment, Parkin knockdown blocked, while overexpression significantly enhanced, the reduction of SFXN2 levels (Figures 8C,D), suggesting that Parkin also negatively regulates SFXN2 in response to mitochondrial dysfunction in neuronal cells.

Apoptosis analysis via flow cytometry showed that in SH-SY5Y cells, SFXN2 knockdown significantly increased apoptosis, along with elevated levels of apoptosis signalling proteins including cytochrome c, BAX, and cleaved-caspase3 (Figures 8E–G). Conversely, SFXN2 overexpression attenuated CCCP- and MPP^+^-induced apoptosis in SH-SY5Y cells (Figures 8H–K; Supplementary Figure S4). Similar results were observed in Neuro-2a (N2a) mouse neuroblastoma cells, where mSFXN2 knockdown (confirmed via RT-PCR due to the lack of specific antibodies) increased apoptosis, and mSFXN2 overexpression mitigated apoptosis in CCCP- and MPP^+^- treated N2a cells (Supplementary Figures S5A–K).

Collectively, these findings suggest that reduced SFXN2 contributes to neuronal apoptosis resulting from mitochondrial dysfunction, underscoring its potential relevance in neurodegenerative disease pathologies.

## 4 Discussion

Recent studies have shown that SFXN2 is intricately involved in mitochondrial functions, including iron metabolism, heme biosynthesis, bioenergetics, and redox homeostasis (Mon et al., 2019; Chen Y. et al., 2022). However, how SFXN2 is regulated under mitochondrial dysfunction remains unclear. In this study, we provide novel evidence that Parkin mediates SFXN2 degradation via the UPS in response to mitochondrial damage. Functionally, SFXN2 has anti-apoptosis effect, and SFXN2 reduction contributes to mitochondrial damage-induced cell death in various cell lines, including 293 T, SH-SY5Y, and N2a cells. Collectively, these findings offer new insights into the regulation of SFXN2 in mitochondrial dysfunction and highlight SFXN2’s potential implications in neurodegenerative diseases, particularly PD.

Unlike other SFXN proteins on the IMM, SFXN2 is proposed to reside on the OMM. In general, OMM proteins play crucial roles in mitochondrial functions, including the control of mitochondrial quality, mediation of mitochondrial dynamics, maintenance of ion hemostasis, and transport of metabolites and ions (Tan and Colombini, 2007; Xian and Liou, 2021; Chen et al., 2023). In reciprocal, OMM proteins are tightly regulated by mitochondrial status and, in turn, relay signals of mitochondrial dysfunction to various cellular processes (Chen et al., 2023). A well-known example is that mitochondrial depolarization triggers rapid polyubiquitination and degradation of OMM protein Mcl-1, and subsequently, reduced Mcl-1 enhances apoptosis via Bax/Bak channel activation (Carroll et al., 2014). Similarly, our study demonstrates that SFXN2 undergoes negative regulation under mitochondrial stress, and SFXN2 reduction contributes to mitochondrial damage-induced cell death across multiple contexts. These findings highlight SFXN2’s critical role in maintaining cellular viability under mitochondrial stress.

Parkin, a ubiquitin E3 ligase encoded by the *PARK2* gene, is the primary genetic factor in juvenile- and early-onset PD (Kitada et al., 1998). During mitochondrial stress, Parkin is recruited to damaged mitochondria, where it ubiquitinates OMM proteins for proteasomal degradation (Narendra et al., 2008). So far, SFXN2 has not previously been linked with to Parkin’s function, and our study is the first to demonstrate that SFXN2 is a substrate of Parkin-mediated UPS degradation, particularly under mitochondrial dysfunction.

Since we observed reciprocal co-immunoprecipitation of exogenously and endogenously expressed Parkin and SFXN2, while no detectable interaction was observed between SFXN2 and the known Parkin substrate BAX, we believe that Parkin and SFXN2 directly and specifically interact with each other. However, given the complexity of ubiquitin signaling, the involvement of additional adaptor proteins or indirect interaction cannot be entirely excluded. It is also important to note that SFXN2 degradation under mitochondrial stress is likely regulated through multiple pathways. While treatment with the proteasome inhibitor MG132 significantly attenuated CCCP-induced degradation of SFXN2, it only partially restored its protein levels. This observation prompted us to explore lysosomal involvement. Treatment with chloroquine (CQ), a well-characterized lysosomal inhibitor (Mauthe et al., 2018), similarly mitigated SFXN2 degradation under mitochondrial depolarization (Supplementary Figure S6), indicating that lysosome-mediated pathways also contribute to SFXN2 turnover. These results support the notion that additional regulatory mechanisms, both ubiquitin-dependent and -independent, likely contribute to SFXN2 turnover during mitochondrial stress.

Interestingly, HA-Ub overexpression alone does not induce SFXN2 reduction. We speculate that, with excessive amount of HA-Ub, Parkin (or possibly other E3 ligases) could promote K63 linked or other lysine dominated ubiquitination, resulting in stabilization or aggregation of SFXN2, since similar phenomenon has been observed in previous studies with other proteins (McKeon et al., 2015; Peng et al., 2017). Furthermore, in HeLa cells lacking endogenous Parkin, we still detected appreciable levels of SFXN2 polyubiquitination, implying that other E3 ligases may also target SFXN2 for degradation. Unlike many E3 ligases that exhibit strict substrate specificity, Parkin has been shown to ubiquitinate a wide array of mitochondrial proteins during stress, with substrate selection likely influenced by contextual ubiquitination signals rather than specific sequence motifs (Durcan et al., 2012; Rose et al., 2016; Martinez et al., 2017; Koyano et al., 2019). Supporting this notion, the extent of SFXN2 downregulation induced by Parkin overexpression was more modest than that achieved by siRNA-mediated knockdown, suggesting that Parkin may serve to fine-tune, rather than completely abolish, SFXN2 expression.

The regulation of SFXN2 by Parkin also adds complexity to their roles in regulating cellular response to mitochondrial damage. Parkin-mediated ubiquitination events are generally considered cytoprotective, facilitating mitophagy and removing pro-apoptotic signals BAX, BAK, and AIF (Johnson et al., 2012; Guida et al., 2019; Cheng et al., 2023). Contrarily, our findings suggest a pro-apoptotic role for Parkin, as its ubiquitination and degradation of SFXN2 contribute to mitochondrial damage-induced cell death. Interestingly, other studies have also reported pro-apoptotic roles for Parkin under certain conditions. As above mentioned, Parkin promotes cell death by targeting the pro-survival protein Mcl-1 for degradation and enhances apoptosis in TNF*α*-treated cells (Lee et al., 2012; Carroll et al., 2014). These findings, along with ours, suggest that parkin plays a dual role in apoptosis regulation, with SFXN2 acting as a potential molecular switch between its divergent functions. Furthermore, given that Parkin also targets other PD-related proteins, such as α-synuclein and Synphilin-1, we propose that SFXN2 degradation may play a modulatory, rather than central, role in regulating apoptosis and neurodegeneration.

Although the precise mechanism by which SFXN2 regulates apoptosis remains incomopletely defined, prior research in multiple myeloma cells demonstrated that SFXN2 overexpression markedly attenuated EBSS-induced autophagy (Chen L. et al., 2022). In addition, SFXN2 knockdown has been shown to elevate mitochondrial reactive oxygen species (ROS) levels, reduce mitochondrial membrane potential, promote permeability transition pore (PTP) opening, and facilitate cytochrome c release (Chen L. et al., 2022). These mitochondrial perturbations likely trigger Bax translocation and activate downstream apoptotic pathways. Therefore, SFXN2 likely functions as an anti-apoptotic factor by preserving mitochondrial homeostasis, while its loss promotes apoptotic signaling. Moreover, SFXN2 deficiency induces mitochondrial iron overload, disrupts heme biosynthesis, and impairs mitochondrial respiration (particularly affecting complexes II–IV), resulting in compromised oxidative phosphorylation and increased susceptibility to iron-induced oxidative stress (Mon et al., 2019). These defects may predispose cells to ferroptosis or other iron-dependent cell death pathways (Gao et al., 2015; Mon et al., 2019). Collectively, these studies position SFNX2 as a key regulator of mitochondrial integrity and cell fate.

In addition, this study suggests a connection between SFXN protein function and PD. While the role of SFXN proteins in human health remains largely unexplored, emerging data start to link SFXN proteins to mitochondrial dysfunction-related diseases. For example, loss-of-function mutations in *SFXN4* are associated with a rare mitochondrial disease characterized by mitochondrial complex I deficiency (Hildick-Smith et al., 2013; Sofou et al., 2019). SFXN1 and SFXN3 are downregulated in both Alzheimer’s disease and PD, both conditions marked by mitochondrial dysfunction (Simunovic et al., 2009; Minjarez et al., 2016). Additionally, recent GWAS studies have pointed a potential association between SFXN2 and PD (Simon-Sanchez et al., 2009; Li et al., 2021). Here, we demonstrate that SFXN2 is a direct substrate of Parkin, whose dysregulation contributes to both inherited and sporadic PD, and overexpression of SFXN2 provides protects neuronal cells from mitochondrial damage-induced apoptosis. Interestingly, in *Drosophila*, *dSFXN1/3* overexpression has been reported to protect dopaminergic neurons from death (Amorim et al., 2017). Together, these findings suggest that SFXN proteins play important role in mitochondrial dysfunction-related disease like PD.

In conclusion, SFXN2 is identified as an important regulator of cellular responses to mitochondrial damage. Under these conditions, Parkin-mediated ubiquitination negatively regulates SFXN2, promoting mitochondrial damage-induced apoptosis. Further research is needed to elucidate the detailed mechanisms of SFXN2’s anti-apoptotic effect and to explore whether modulating SFXN proteins could be a viable neuroprotective strategy for neurodegenerative diseases.

## Data Availability

The original contributions presented in the study are publicly available. RNA-seq data used in this study has been uploaded to the NCBI GEO database with accession number GSE294872.
