# Supplementary material for: SFXN2 contributes mitochondrial dysfunction-induced apoptosis as a substrate of Parkin
Source: Front Cell Neurosci. 2025 Aug 14;19:1623747. doi: 10.3389/fncel.2025.1623747 (PMC12391048; doi:10.3389/fncel.2025.1623747)
Supplement: Supplementary file 3 [file Table_3.pdf]

Supplementary Table 3. List of antibodies used in this study

Target Protein	Description	Company Cat no.	Working conc	Experiment used	RRID number
SFXN2	Rabbit polyclonal anti-SFXN2	Novus Biologicals (NBP1-85960)	1:1000	WB	AB_11036956
PINK1	Rabbit Monoclonal anti-PINK1 (D8G3)	Cell Signaling Technology (6946S)	1:1000	WB	AB_11179069
Parkin	Mouse monoclonal anti-Parkin	Santa Cruz Biotechnology (sc-32282)	1:1000	WB	AB_628104
Tom20	Rabbit Monoclonal anti-Tom20	Cell Signaling Technology (42406S)	1:1000	WB/IF	AB_2687663
Myc	Rabbit Monoclonal anti-Myc-Tag (71D10)	Cell Signaling Technology (2278S)	1:1000	WB/IF	AB_490778
HA	Rabbit Monoclonal anti-HA-Tag (C29F4)	Cell Signaling Technology (3724S)	1:1000	WB/IF	AB_1549585
Ubiquitin	Mouse monoclonal anti-Ubiquitin (P4D1)	Cell Signaling Technology (3936S)	1:1000	WB	AB_331292
Cleaved-caspase3	Rabbit Monoclonal anti-Cleaved Caspase-3 (Asp175) (5A1E)	Cell Signaling Technology (9664S)	1:500	WB	AB_2070042
BAX	Rabbit polyclonal anti-BAX	Cell Signaling Technology (2772S)	1:1000	WB	AB_10695870
Cytochrome C	Mouse monoclonal anti-Cytochrome C	Santa Cruz Biotechnology (sc-13156)	1:1000	WB	AB_627385
α-Tubulin	Rabbit Monoclonal anti-alpha Tubulin	Abcam (ab52866)	1:1000	WB	AB_869989
Mouse IgG	HRP-conjugated goat anti-mouse IgG	Jackson ImmunoResearch Labs (115-035-003)	1:10000	WB	AB_10015289
Rabbit IgG	HRP conjugated goat anti-rabbit IgG	Jackson ImmunoResearch Labs (111-035-144)	1:10000	WB	AB_2307391
Rabbit IgG	Goat anti-Rabbit IgG (H+L) Highly Cross-Adsorbed Secondary Antibody, Alexa Fluor™ Plus 488	Invitrogen (A32814)	1:400	IF	AB_2633280

Target Protein	Description	Company Cat no.	Working conc	Experiment used	RRID number
Mouse IgG	Cy™3 AffiniPure™ Goat Anti-Mouse IgG	Jackson ImmunoResearch Labs (115-165-003)	1:400	IF	AB_2338680
Rat IgG	Goat anti-Rat IgG (H+L) Cross-Adsorbed Secondary Antibody, Cyanine5	Invitrogen	1:400	IF	AB_2534034
