# Supplementary material for: SFXN2 contributes mitochondrial dysfunction-induced apoptosis as a substrate of Parkin
Source: Front Cell Neurosci. 2025 Aug 14;19:1623747. doi: 10.3389/fncel.2025.1623747 (PMC12391048; doi:10.3389/fncel.2025.1623747)
Supplement: Supplementary file 5 [file Supplementary_file_1.docx]

**
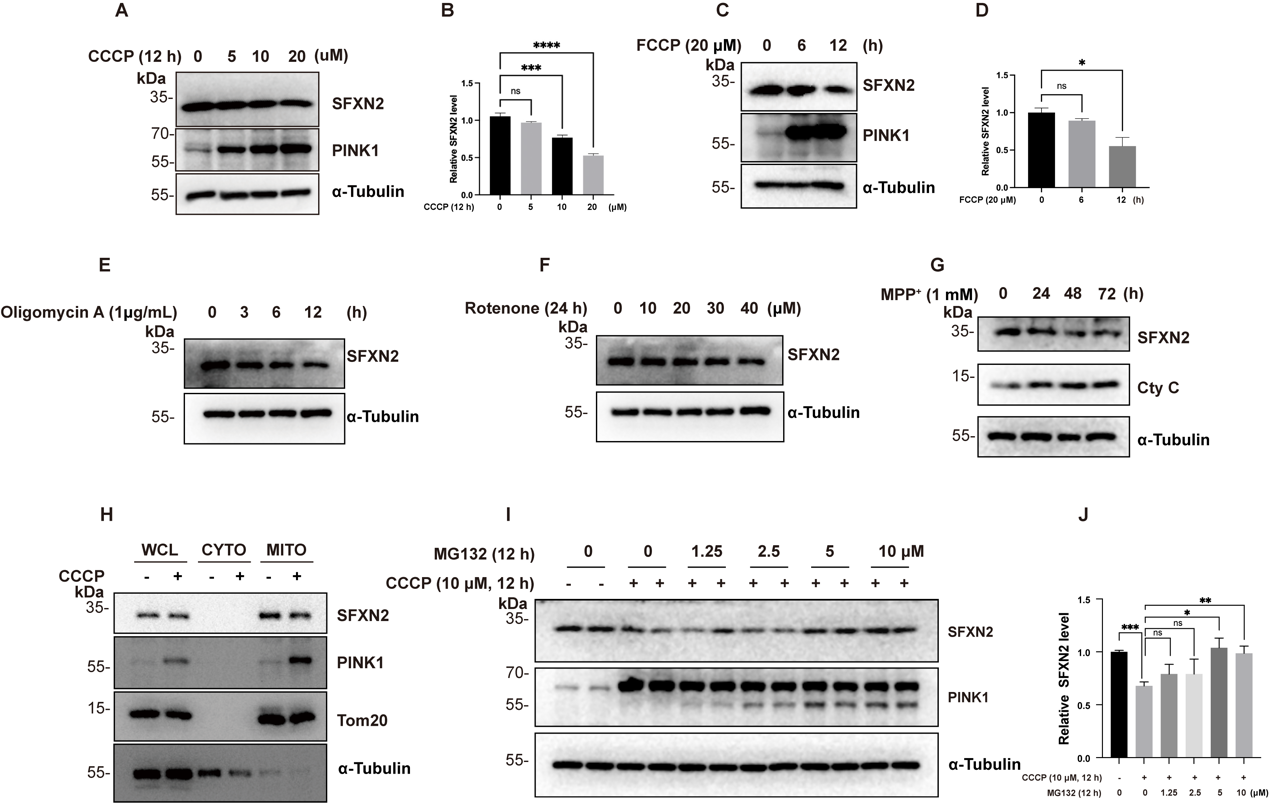
**

**Supplementary Figure 1. Mitochondrial impairments lead to decreased SFXN2 protein levels**

**(A–B)** Western blot (WB) analysis of SFXN2 and PINK1 protein levels in HEK293 cells treated with various concentrations of CCCP for 12 h (**A**). Quantification of the relative SFXN2 protein levels, normalized to α-Tubulin, is presented in the histogram (**B**).

(**C**) WB analysis of SFXN2 and cytochrome c (Cyt C) protein levels in HEK293 cells treated with 1 mM MPP^+^ for the indicated durations.

**(D–E)** WB analysis of SFXN2 and PINK1 protein levels in HEK293 cells treated with 20 μM FCCP for the indicated time periods (**D**), with the relative SFXN2 protein levels normalized to α-Tubulin quantified and presented in the histogram (**E**).

**(F–G)** WB analysis of SFXN2 protein levels in HEK293 cells treated with 1 μg/mL Oligomycin A for the indicated time periods (**F**), or with various concentrations of Rotenone for 24 h (**G**).

**(H)** WB analysis of SFXN2, Tom20 and PINK1 protein levels in the whole cell lysate (WCL), cytosolic (CYTO), and mitochondrial (MITO) fractions prepared from HEK293 cells treated with vehicle control (0.05% (v/v) DMSO) or 20 μM CCCP for 12 h. α-Tubulin and Tom20 were used as loading controls for CYTO and MITO fractions, respectively.

(**I–J**) WB analysis of SFXN2 and PINK1 protein levels in HEK293 cells treated with vehicle control (0.05% (v/v) DMSO), CCCP (10 μM) alone, or CCCP (10 μM) combined with varying concentrations of MG132 for 12 h (**I**). Quantification of relative SFXN2 protein levels normalized to actin is presented in the histogram (**J**).

Images are representative of at least three independent experiments with similar results. Histogram data are presented as mean ± SEM (N = 3). Statistical significance was analyzed by one-way ANOVA. ** p < 0.05*, *** p < 0.01*, **** p < 0.001*, ***** p < 0.0001,* ns = non‑significant.

**
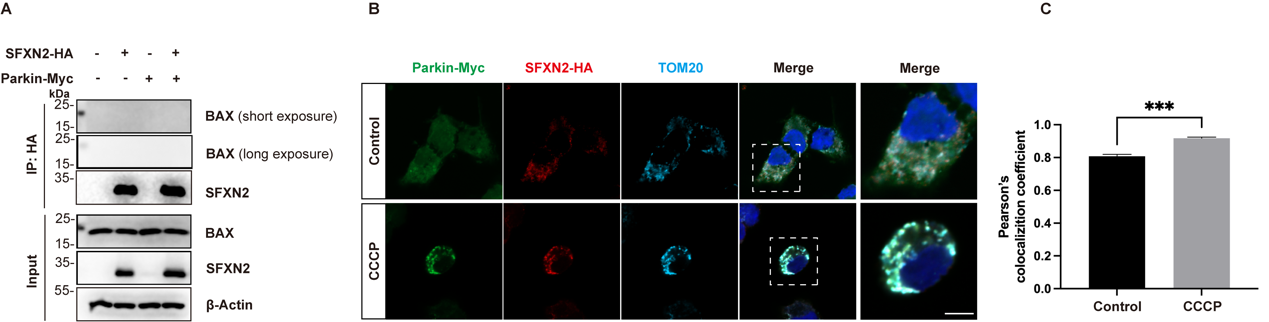
**

**Supplementary Figure 2.** **SFXN2 interacts with Parkin, but not with BAX in HEK293 cells**

**(A)** Western blot analysis of the co‑immunoprecipitation between SFXN2 HA and BAX in transfected HEK293 cells expressing the indicated constructs or vector control. Cell lysates were immunoprecipitated using anti-HA antibody, followed by immunoblotting (IB) with anti-SFXN2 and anti-BAX antibodies. Input lysates were used as controls for the IB.

**(B–C)** Representative immunofluorescent images of HEK293 cells overexpressing Parkin-Myc and SFXN2-HA, treated with either 20 μM CCCP or vehicle control (0.05% (v/v) DMSO) for 6 h. Cells were immunostained with anti‑Myc (green), anti‑HA (red), and anti‑TOM20 (light blue) antibodies. Scale bars, 5 μm. The histogram **(B)** presents the quantification of Pearson's correlation coefficient between Parkin‑HA and SFXN2-Myc.

Images are representative of at least three independent experiments. Histogram data are presented as mean ± SEM (N = 3). Statistical significance was analyzed by Student’s *t*-test. **** p < 0.001*.


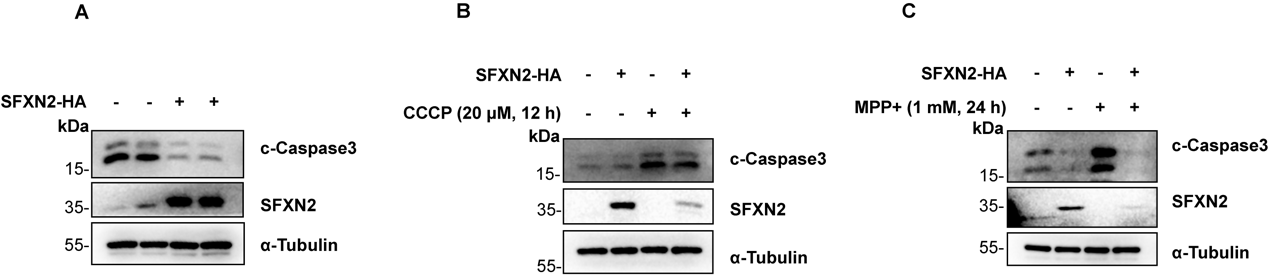


**Supplementary Figure 3.** **Overexpression of SFXN2 protects toward mitochondrial impaired-induced Apoptosis in HEK293 cells**

**(A)** Western blot (WB) analysis of cleaved Caspase3 (c-Caspase3) and SFXN2 protein levels in HEK293 cells transfected with vector control or SFXN2-Myc.

**(B–C)** WB analysis of c-Caspase3 and SFXN2 protein levels in HEK293 cells transfected with vector control or SFXN2-Myc, with the addition of 20 μM CCCP or vehicle control (0.05% (v/v) DMSO) for 12 h (**B**), or 2 mM MPP^+^ or vehicle control (0.05% (v/v) DMSO) for 24 h (**C**).


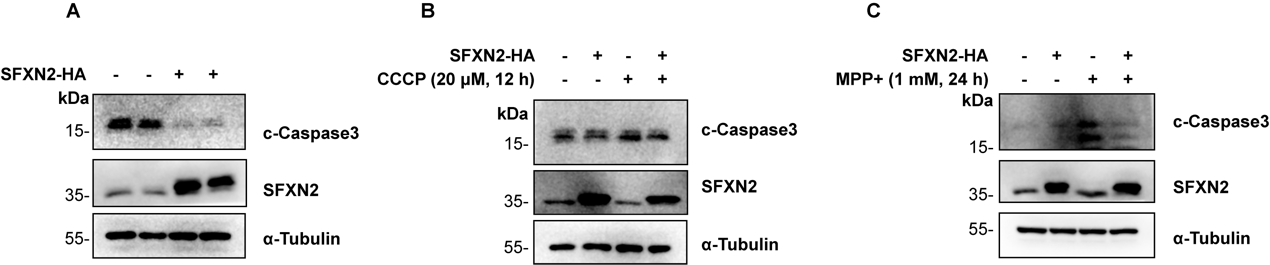


**Supplementary Figure 4. SFXN2 overexpression protects against mitochondrial impairment-induced apoptosis in SH-SY5Y cells**

**(A)** Western blot (WB) analysis of cleaved Caspase3 (c-Caspase3) and SFXN2 protein levels in SH-SY5Y cells transfected with either vector control or SFXN2-Myc.

**(B–C)** WB analysis of c-Caspase3 and SFXN2 protein levels in SH-SY5Y cells transfected with vector control or SFXN2-Myc, with the addition of 20 μM CCCP or vehicle control (0.05% (v/v) DMSO) for 12 h (**B**), or 1 mM MPP^+^ or vehicle control (0.05% (v/v) DMSO) for 24 h (**C**).


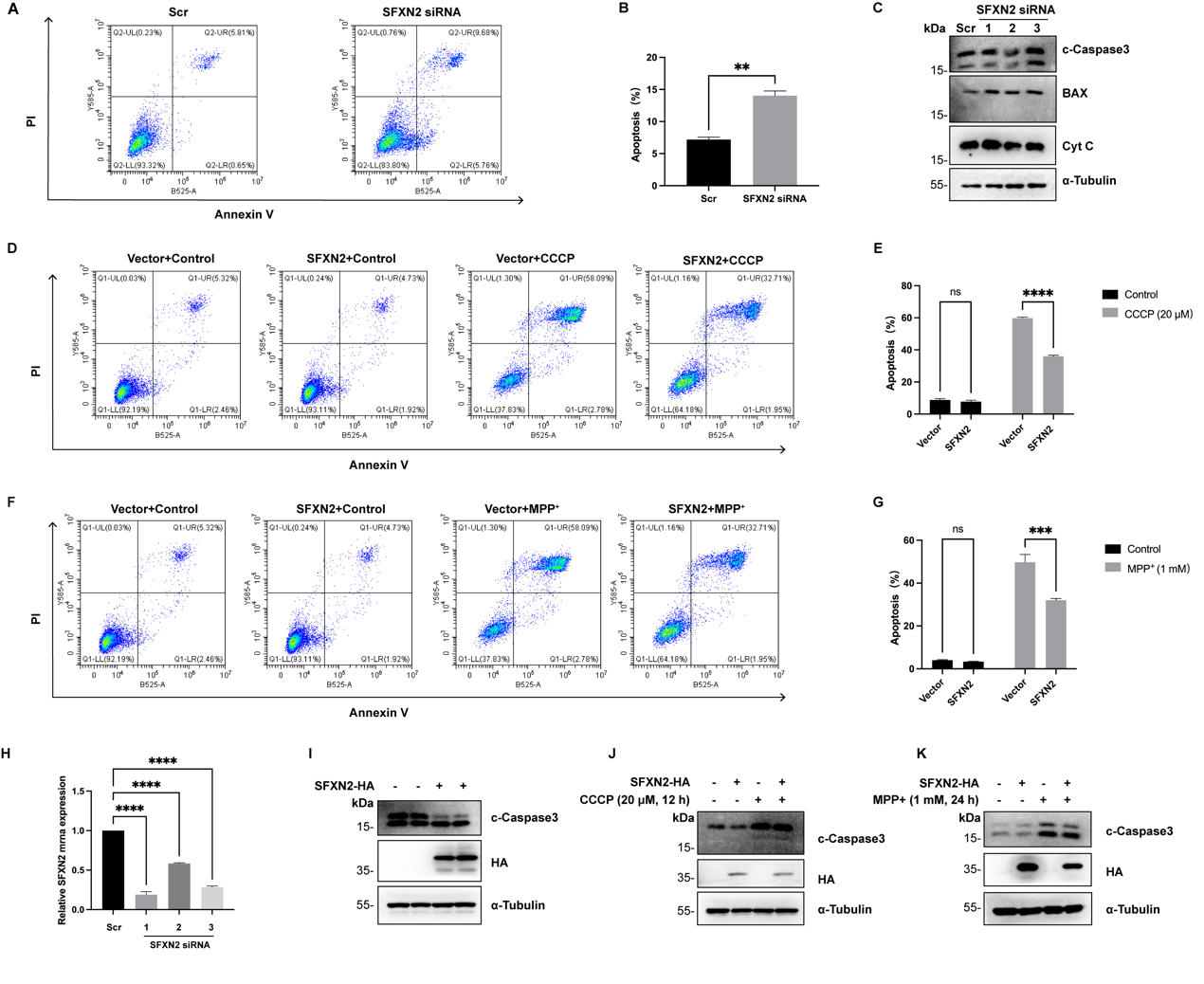


**Supplementary Figure 5. SFXN2 regulates apoptosis in Neuro-2a cells**

(**A–B**) Annexin V/PI staining and flow cytometry assays of Neuro-2a cells transfected with scrambled siRNA or siRNA targeting SFXN2 (**A**), with quantification of the percentage of apoptosis cells (Annexin V‑positive cells) presented in the histogram (**B**).

(**C**) Western blot (WB) analysis of Cyt C, BAX, c-Caspase3, and SFXN2 protein levels in Neuro-2a cells transfected with scrambled siRNA or siRNA targeting SFXN2.

(**D–E**) Annexin V/PI staining and flow cytometry assays of transiently transfected Neuro-2a cells expressing SFXN2 or control vector, followed by treatment with vehicle control (0.05% (v/v) DMSO) or 20 μM CCCP for 12 h (**D**). Quantification of the percentage of apoptotic cells (Annexin V‑positive cells) is presented in the histogram (**E**).

(**F–G**) Annexin V/PI staining and flow cytometry assays of transiently transfected Neuro-2a cells expressing SFXN2 or control vector, followed by treatment with vehicle control (0.05% (v/v) DMSO) or 1 mM MPP^+^ for 24 h (**F**). Quantification of the percentage of apoptotic cells (Annexin V‑positive cells) is presented in the histogram (**G**).

**(H)** Quantitative PCR analysis of mouse SFXN2 mRNA levels in Neuro-2a cells transfected with scrambled siRNA or SFXN2‑targeting siRNA.

**(I–K)** WB analysis of cleaved Caspase3 (c-Caspase3) and SFXN2 protein levels in Neuro-2a cells transfected with either vector control or SFXN2-Myc, under basal conditions (I), or following treatment with 20 μM CCCP or vehicle control (0.05% (v/v) DMSO) for 12 h (**J**), or treatment with 1 mM MPP^+^ or vehicle control (0.05% (v/v) DMSO) for 24 h (**K**).

Images are representative of at least three independent experiments. Histogram data are presented as mean ± SEM (N = 3). Statistical significance was analyzed by two‑way ANOVA. *** p < 0.01*, **** p < 0.001, **** p < 0.0001*, ns = non‑significant.


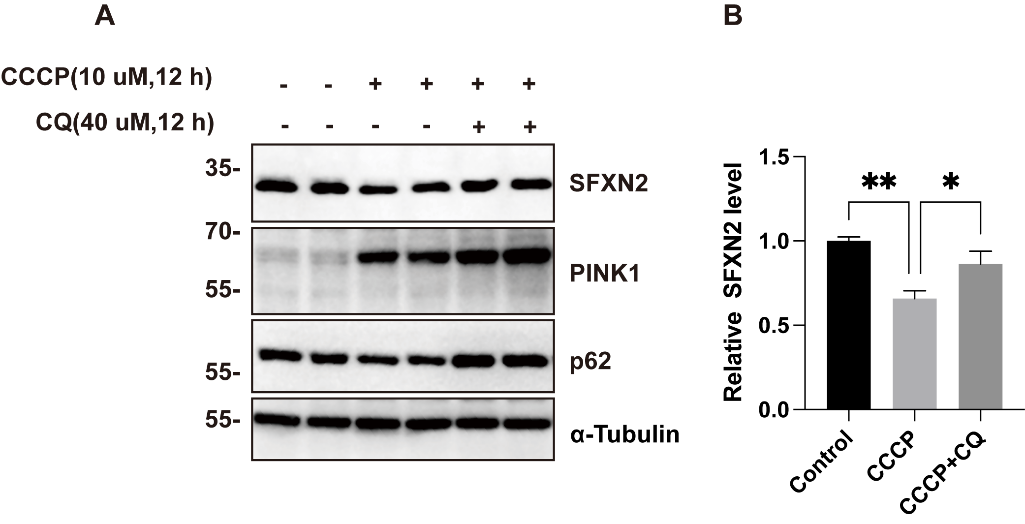


**Supplementary Figure 6. Chloroquine (CQ) treatment partially restores mitochondrial dysfunction‑induced SFXN2 reduction**

(**A–B**) Western blot analysis of SFXN2, P62 and PINK1 protein levels in HEK293 cells treated with vehicle control (0.05% (v/v) DMSO), CCCP (10 μM) alone, or CCCP (10 μM) combined with chloroquine CQ (40 μM) for 12 h (**A**). Quantification of relative SFXN2 protein levels normalized to actin is presented in the histogram (**B**).

Images are representative of at least three independent experiments with similar results. Histogram data are presented as mean ± SEM (N = 3). Statistical significance was analyzed using one-way ANOVA. ** p < 0.05, ** p < 0.01*.
